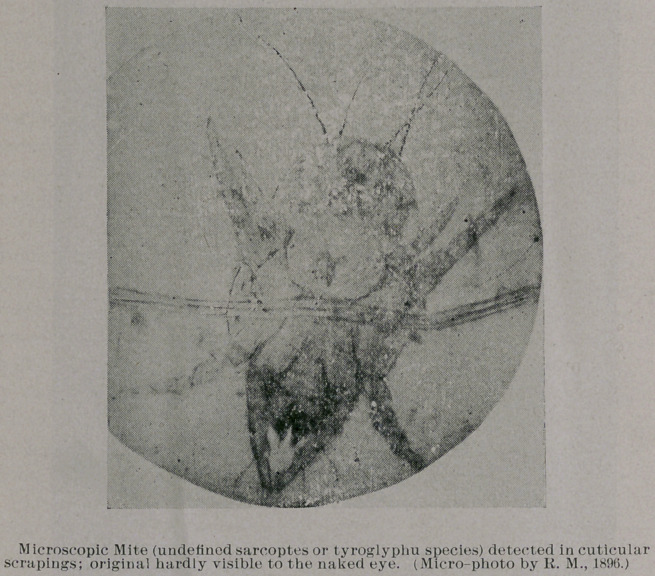# Photo-Micrography as Related to Medical and Scientific Research*Original paper read before the annual meeting of the Western Texas Medical Association, by Dr. Wm. Luter, Secretary, October 27, 1898, and published in the Medical Era, April, 1899.

**Published:** 1899-06

**Authors:** Rudolph Menger

**Affiliations:** San Antonio, Texas


### TEXAS MEDICAL JOURNAL.

ESTABLISHED JULY. 1885.

PUBLISHED MONTHLY.—SUBSCRIPTION $1.00 A YEAR.

VOL. XIV. AUSTIN, JUNE, 1899. No. 12.

### Original Contributions.

Revised and enlarged for Texas Medical Journal.

### Photo-Micrography as Related to Medical and Scientific Research.*

BY RUDOLPH MENGER, M. D., SAN ANTONIO, TEXAS.

Having had many years of practical experience in comparative photo-micrography, as applied to microscopic as well as medical and scientific research, I take great pleasure in giving in the following papers some of my own views in this especial line of photography, illustrated by a collection and preparation of my own of over two hundred photo-micrographs of the most diverse microscopical material.

It is not my intention to go into historic details concerning past and modern microscopy as such; this is more or less known; but it will be my endeavor to give a clear résumé of practical photo-micrography, such as is of interest to the general practitioner as an accessory to microscopic work.

The enormous advancement in all branches of science and art, and particularly also in modern microscopic research, has solved problems which in former years appeared to be miracles or illusions. Not alone with our compound and improved microscopes are we able to detect even the minutest of cells or tissues and organisms, but, as you know, we can reproduce and copy in the minutest of de-

*Original paper read before the annual meeting of the Western Texas Medical Association, by Dr. Wm. Luter, Secretary, October 27, 1898, and published in the Medical Era, April, 1899.

tails the very image of those same and otherwise invisible objects by means of the combined photo-micrographic process.

Therefore, whatever microscopic objects of the finished slide the powerful lenses of the microscope reveal to the retina of the eye, they can be permanently photographed, and by such means the histological appearance of any given microscopic object can be illus-

PLATE I.—Blood corpuscles of man (a) and pigeon (b) partly isolated and coagulated. Lamp light exposure: 6 min. Examine also with a magnifying glass. x :ab. 80. (Micro-photo by R. M., 1898.)

trated, from the crude microscopic slide up to many hundred and even thousand times especially magnified.

In anatomical and pathological histology, urinalysis, bacteriology, crystallography, and various allied sciences, photo-micrography goes hand in hand with microscopy as a means of detection, analysis, and preservation; but the real value of the latter seems hardly enough appreciated. By its aid, for instance, in cellular histology, and more particularly in hæmatology and bacteriology proper, a much wider microscopic field of the diameter of a given specimen can be permanently reproduced than by microscopic examination alone, and, besides, it can always be examined at leisure as to its clinical and scientific value.

Here, for instance, on five of the especially prepared photo-micrographs of blood-preparations, the photos signed "I.," "II." and "III." show a minute particle of a drop of human blood and blood of pigeon which had been gathered on the same slide glass.

The diameter of the micrographic field of two of these photo specimens, as in all others of my collection, shows a circumference of four by five inches, whilst in the original microscopic field, without the aid of the camera lenses, the width of the microscopic field is only about three inches; and in plate three, showing blood corpuscles of man, magnified about six hundred times, the photo-micrographic field is even eight by ten inches in diameter. The latter (which has been reduced in the annexed illustration (Plate II.) to the size of five by six inches) is rather an unusually interesting specimen from a scientific point of view. It includes in its diameter, by actual count (by dividing its diameter into twenty-five cubic sections) a total of about one thousand and fifty blood corpuscles, corresponding to about nine thousand two hundred of blood cells to a drop of blood thinly spread out on the slide glass from which it was photographed. This number of corpuscles of one thousand and fifty is of course only approximately correct, but it is as near correct as the cells can be distinguished and counted from the conglomerated cell-masses inclosed in the photo-micrographic field.

Referring again to the photo plates I., II. and III. (which are somewhat reduced from the original diameter), it will be seen what can be accomplished in photo-micrography for comparative purposes. All of these three micro-photos represent, as object lessons, a *minute particle of a drop of blood of man and pigeon,* mounted near each other on one and the same slide glass. Plate I. shows the corpuscles isolated, conglomerated or in the shape of coagula, and in the upper right side the nucleated corpuscles of pigeon are seen plainly, and which will show up better if seen extra through a magnifying glass. Plate III. represents a portion of the same nucleated cells as seen on plate I., very highly magnified—nearly two thousand times, as near as I can judge the same, approximately; and plate II. shows, as stated above, the corpuscles of man, also highly magnified, under electric light exposure. (I prepared the two latter photos with especial appliances, after elongating and applying different diagrams to the tube adjustment of the micro-

PLATE II.—Blood corpuscles of man (the cells slightly shriveled), representing about one-sixth part of a drop of blood, from the same specimen of blood seen on Plate I. Magnified about 600. Photo made with electric light exposure: 14 min., with improved microscope. (Micro-photo by R. M. and E. Raba, 1898.)

scope. The original size of Plate III., eight by ten inches, was prepared by a local photographer with one of his large-sized cameras, shown in the *Era* and the TEXAS MEDICAL JOURNAL, are all my own work.

The main point of this work of *high-power micro-photography* is to get sufficient light through the many lenses of the microscope and camera in order to see and sharply to focus the objects on the background glass. Both is, in many instances, a difficult task, and it takes a great deal of practical experience to reproduce the objects on the slide glass intended for preservation. I use no oil immersion in this work.

Much also depends on the kind of light used. For high-power examination electric light is, of course, the best, but even for such delicate work lamp light is sufficient, provided it is properly applied and concentrated by the aid of an especially thick condensing lens. My entire work in this line has been done by the aid of lamp light, which, in very high-power examination, takes some seconds or minutes longer, but accomplishes as good results as if electric light had been used, depending upon the proper time of exposing the sensitized plates.

Photo-micrography should always be done at night-time, when the surroundings are quiet (as the least vibration of the microscope during the process of photo-exposure spoils the ultimate result), and when the immense sensitized plates can be best handled in exchanging them to and from the plate holder, or during the process of developing; and, besides, the light of the bull's-eye lens thrown upon the microscopic objects is more intense when the surroundings are made entirely dark and the light-rays of the condensing lens are then extra concentrated. And it is an interesting fact that, whilst the most extreme sensitized plates—the so-called flash-light plates—can and are used for such work, but preferably the less sensitized plates, and especially the so-called isochromatic plates, it may take, according to the magnifying power and the condition of the prepared slide, from one to ten or even twenty minutes exposure to produce a sharp likeness of the objects on the slide glass, while it would require only a second and even less time to make a photo-exposure with the same plate in general photographing.

Plate IV. is quite a rare and interesting specimen of trichinosis in man, highly magnified, from a soldier who died from trichinosis many years ago at Austin, Texas, and the mounted specimen was prepared by the late William Barbeck, of San Antonio. It shows a normal stray trichina coiled up and situated near an encapsulated or calcified trichina spiralis, along the muscle tissues.

Plate V. represents bacilli and cocci (spores) in blood of an animal afflicted with glanders (exposure of plate twelve minutes; x: ab. 1200). The specimen contained bacteria of different types, especially also streptococci, but also a large number of bacilli, in shape and description of author's, resembling glander bacilli—*i. e.*, rods somewhat shorter, but thicker than tubercule bacilli; mainly straight and slightly curved, isolated or in groups of quite uniform size and diameter. Some of the bacilli in above plate were extra outlined on the negative in order to make a good print for the photo-

PLATE III.—Nucleated blood corpuscles of pigeon, from same blood-sample as shown on Plate I., representing about one-tenth part of a drop of blood. Lamp light exposure: 18 min, x: ab. 2000. (Micro-photo by R. M., 1898.)

engravure. The bacilli had been stained according to Loeffler's method. The animal in this case, as in a number of others, of which a particle of blood had been furnished by Drs. Lange and Burby, veterinary surgeons, showed all physical and anatomical signs and lesions of glander disease; besides, in all cases, the doctors tell me, the malleine test applied gave a decided reaction on all of the animals. Two of the animals had died, and post-mortem examinations also showed all signs of glander infection. Dr. Souvignet, of our city, is also giving the matter a very close examination in his bacteriological laboratory.

Plate VI. represents a very rare and, on diligent inquiry in literature, an uncatalogued microscopic mite, either of the *tyroglyphi* or *sarcoptes species* (acarinæ). I detected this human parasite

PLATE IV.—Trichinosis: one stray muscular trichina, coiled up, and one encapsulated specimen (lime cystus), both imbedded along the muscle fibrillæ. Highly magnified; original hardly visible. (Micro-photo by R. M., 1898.)

in 1896, in cuticular scrapings from an aged person who was afflicted with a very peculiar cutaneous disease, according to Dr. Flemming, of Georgetown, Texas. Dr. Fleming at the time kindly favored me with the following history of his patient:

"About eight months ago my patient became afflicted with the disease an has been a great sufferer ever since. The disease appears with small papules here and there, from a pale to a fiery red, and at times under treatment will seen apparently well, but on application of ointments or lotions reappear in greater or less number and larger or smaller lesions. The disease is not attended with itching, but when very red has a slight burning sensation. The animaculæ, it seems, on maturing, emerge from the skin, and in some places seem to discharge germs covering a space more or less dense from a half to two inches in diameter. The various remedies I have used have caused many of all sizes to come to the surface; some bore under the skin again; and although I have picked off thousands, I have never seen one move. One of the great annoyances of the patient is their crawling on the skin. Their bite is much like that of a flea or a chinch, and often so rapid is it done that the mite will bore in before you can pick it off with the point of a knife. The bites and pimples never suppurate nor exude serum.

"I have given six months of study and investigation to the disease, and have found nothing in our medical literature which at all resembles it. I have carefully watched him, so that he could not deceive me nor any one else. Besides, he is too anxious to get well for a malingerer.

"I assure you that every particle of the samples I sent you came from his body—he never has taken a sand bath; he always washes himself in hot water, as it seems to give him more relief. I have watched him closely for six months, and have tried every known remedy with no success. The particles of sand-like material or shells, or whatever it is, all come from him, and are not put on him by washing or any application. When I use vinegar on him there will come out on his body more sand or shells; and in the morning his body contains more than in the day-time, keeping him awake through the night, which annoys him so he rests barely at night. I have scraped regular barnacles formed by the insects at night from between his toes and creases of the arms and elbows," etc.

This mite is of the size of the common itch-mite, hardly visible to the noked eye, of yellowish-brown color, supplied with eight legs, five jointed, and the pedal extremities are supplied with a sucking disk—characteristic of the sarcoptes or itch-parasites. The eight legs are decidedly thoracic, not marginal, and the specimen preserved was a *male* one—the sex found being considered by experts as of very rare occurrence. In comparing this mite with the common cheese-mite and fruit acari, our mite shows the legs, jaws, abdomen and bristles more fully developed, the latter closer to the base of the abdomen, larger and thicker.

As seen on the photo-reproductions, I succeeded in making several micro-photos of the parasite in different stadia, and also of the larva. The latter is six-legged; the body and legs were semi-transparent and dotted throughout. I have not encountered any such larva in microscopic mites before. The Smithsonian experts also declared it to be the larva of the parasite under question.

Professor Allen Smith, of the Galveston (Texas) University, in October, 1896, had given me a very interesting report on sarcoptic mites in general, and of our acarus in particular, and I only include here the following points: "I have been looking up all the

PLATE V.—Bacilli and cocci (spores) in blood-sample of animal infected with glanders. x: ab. 1200. (Micro-photo by R. M., 1898.)

data I can get hold of in my endeavor to identify the dermal parasite. There seems to me to be no doubt of the parasite being an acarus. The mode of articulation of its legs, the fact of its having five divisions to each limb, its choliform or pincers-like jaws, in my mind place surely among the sarcoptides. (Here follows an exhaustive explanation of the five tribes of the sarcoptes family, having used as guide, *Meguin:'Les Parasites articules.'*) The five tribes are: *Sarcoptes detricoles, S. plumicoles, S. cysticoles, S. glicicoles* and *S. sporae*. . . . The specimen in hand cannot belong to the first tribe. It differs in being provided with a somewhat rugous integument, in having unequal limbs, and, I believe, dissimilar in having a distinct cleft in the abdominal extremity. It is not to be mixed up with the bird-infesting sarcoptes (*S. plumicoles*)—the latter has all its legs well developed, and never even

Microscopic Mite (undefined sarcoptes or tyroglyphu species) detected in cuticular scrapings; original hardly visible to the naked eye. (Micro-photo by R. M., 1896.)

tending to the abortive (as in the last pair of the parasite), and never produce painful or itching sensations (by some poison in its bite). . . . I would place R.'s parasite, from its shape, its somewhat striated coat, its undeveloped hind pair of legs, and its power to produce itching, among the true itch-sarcoptides."

I have compared our parasite with a number of mites of old fruit and cheese, and it differs in being a smaller and "bolder" appearing acarus, and the endpads of the legs, on high-power examination, showing a stirrup-shaped *discus* or *sucking-cup*. This distinguishes the sarcoptes genus from similar acari. In micro-photography, of course, only such objects and outlines can be copied as come under sharp focus of the lenses of the microscope, especially in making a micro-photo copy of such a minute object as our mite under a very high magnifying power. For this reason the outlines of the terminal parts (sucking-cup) of the legs are not so sharply outlined as the rest of the parasite's body.

This case of parasitic disease seems to be unique in many particulars regarding etiology and symptomatology. With the exception that it was noticed over nearly the entire body, the symptoms, as stated by Dr. Flemming, would about tally with those of the *common itch sarcoptes* of man; but, as noticed, had patient the usuar itch-plague, there certainly would have been found remnants of the itch-parasite and its larva, ova, etc.; and then the itch disease is easily amenable to rigid antiparasitic treatment. The specimens, or remnants from scraping of the skin sent to me were, of course, in a dried-up state; they formed a yellowish-brown, granular powder, showing, on examination, numbers of cuticular and more deeply-seated remnants, capilli (sparingly), calcareous remnants, some granular (apparently hemorrhagic) detritus, shed skins of microscopic mites, and the parasites, either entire, but contracted, or in remnants (partly incrustated, it seemed). The latter were *dead acarinae,* and the one specimen, now under question and illustrated, had its legs contracted when first found, but, under cover-glass pressure, the legs were gradually spread out.

In conclusion, I beg to call attention to the fact that an article on this subject has been published some time before. I had sent the article (in pamphlet form) to a large number of experts and medical institutions here and in Europe, and received from none any data concerning a similar parasite except the Smithsonian experts, who cited a few instances in literature of parasitic mites of the tyroglyphi type found (accidentaily, it seems) in ulcerated cuticular lesions, etc. The authorities of the Zoölogical Institute of Genoa, Italy, have sent me, in return, some literature on microscopic mites, with illustrations, but no such mite is mentioned. Whether, in our case, the parasites had been implanted accidentally on the patient from some animal infested with sarcoptic disease, or from some other unknown source, of course can only be conjectured; but the fact remains that the entire case, as above described, is unique, and the parasites found are some *uncatalogued sarcoptes species*—in my own humble opinion, at least.

San Antonio, Texas, May, 1899.

## Figures and Tables

**Plate I. f1:**
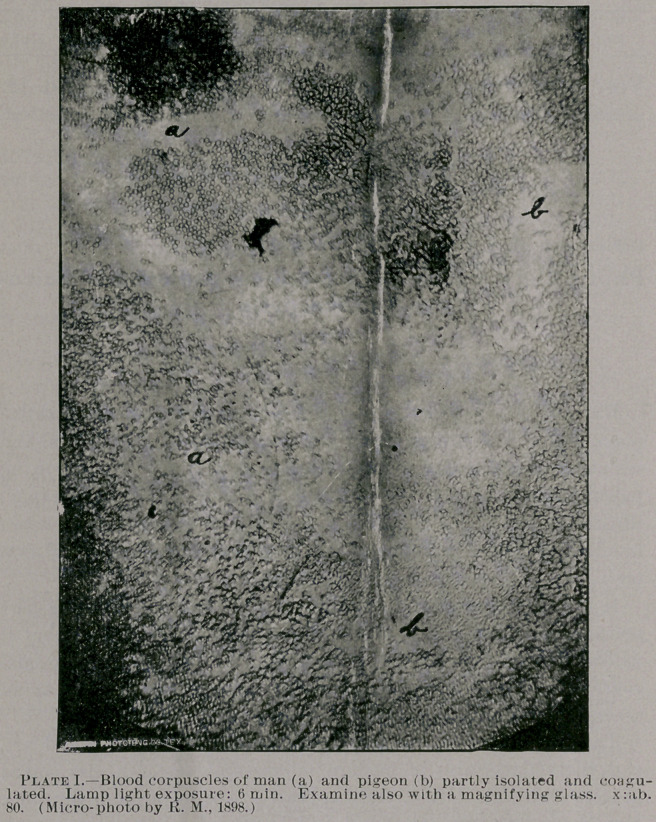


**Plate II. f2:**
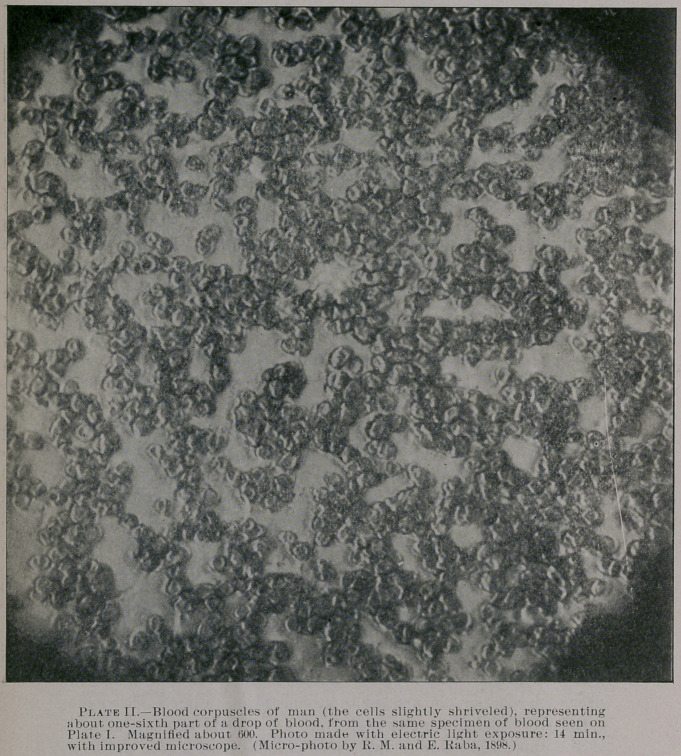


**Plate III. f3:**
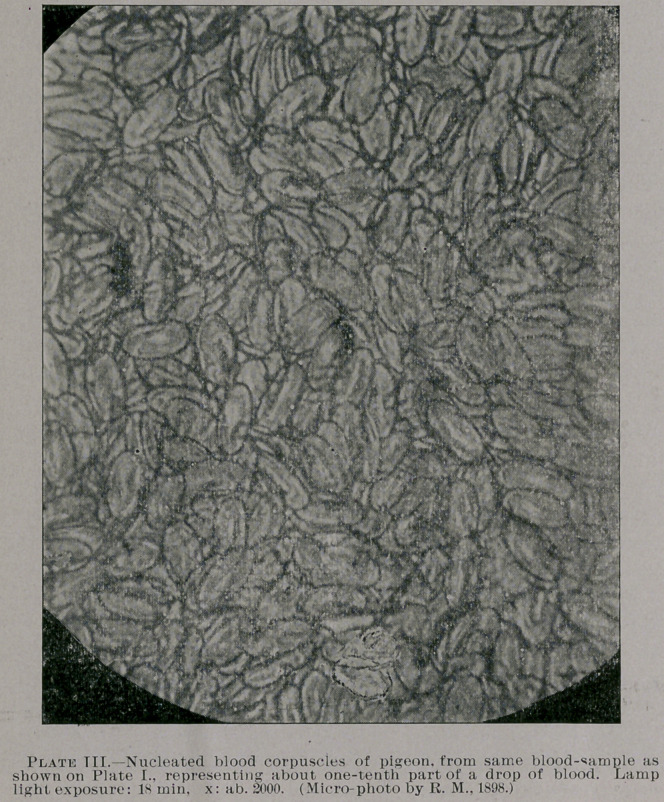


**Plate IV. f4:**
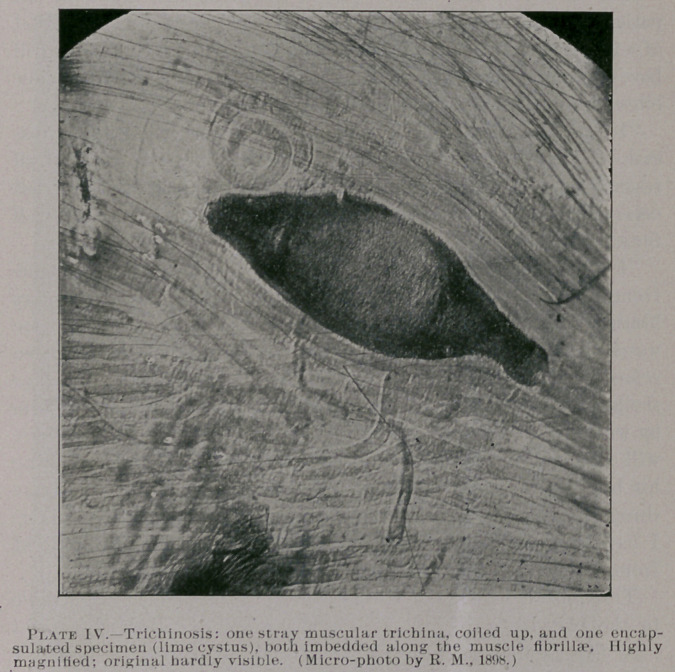


**Plate V. f5:**
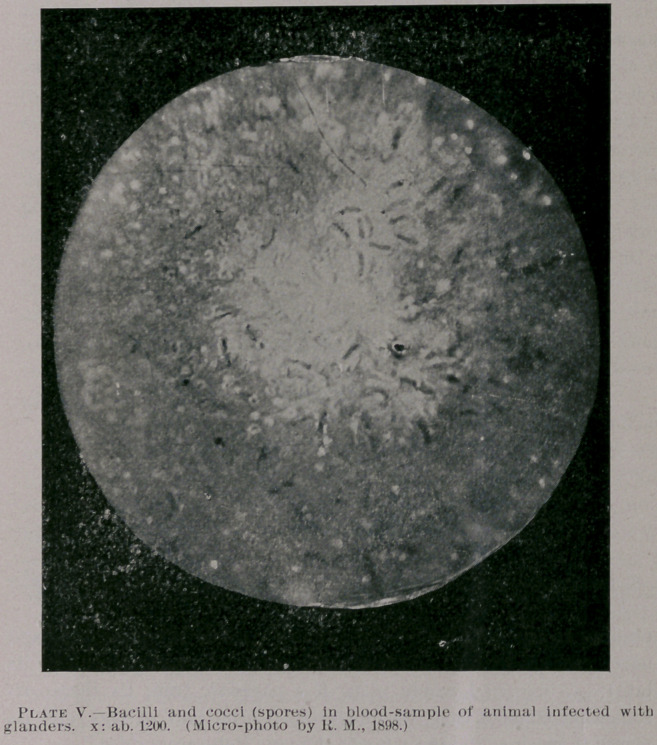


**Figure f6:**